# Microbial Diversity and Mineralogical-Mechanical Properties of Calcitic Cave Speleothems *in Natural* and *in Vitro* Biomineralization Conditions

**DOI:** 10.3389/fmicb.2018.00040

**Published:** 2018-02-02

**Authors:** Navdeep K. Dhami, Abhijit Mukherjee, Elizabeth L. J. Watkin

**Affiliations:** ^1^Biologically Activated Materials Laboratory, Department of Civil Engineering, Curtin University, Perth, WA, Australia; ^2^School of Biomedical Sciences, Curtin Health Innovation Research Institute-Biosciences, Curtin University, Perth, WA, Australia

**Keywords:** speleothem, biomineralization, microbial diversity, nanoindentation, calcium carbonate precipitation, mineralogy

## Abstract

Natural mineral formations are a window into important processes leading to carbon storage and mineralized carbonate structures formed through abiotic and biotic processes. In the current study, we made an attempt to undertake a comprehensive approach to characterize the mineralogical, mechanical, and microbial properties of different kinds of speleothems from karstic caves; with an aim to understand the bio-geo-chemical processes in speleothem structures and their impact on nanomechanical properties. We also investigated the biomineralization abilities of speleothem surface associated microbial communities *in vitro*. Mineralogical profiling using techniques such as X-ray powder Diffraction (XRD) and Tescan Integrated Mineral Analyzer (TIMA) demonstrated that calcite was the dominant mineral in the majority of speleothems with Energy Dispersive X-ray Analysis (EDS) indicating a few variations in the elemental components. Differing proportions of polymorphs of calcium carbonate such as aragonite and vaterite were also recorded. Significant variations in trace metal content were recorded through Inductively Coupled Plasma Mass Spectrometer (ICP-MS). Scanning Electron Microscopy (SEM) analysis revealed differences in morphological features of the crystals which varied from triangular prismatic shapes to etched spiky forms. Microbial imprints and associations were seen in a few sections. Analysis of the associated microbial diversity showed significant differences between various speleothems at Phylum level; although Proteobacteria and Actinobacteria were found to be the predominant groups. Genus level microbial associations showed a relationship with the geochemistry, mineralogical composition, and metal content of the speleothems. The assessment of nanomechanical properties measured by Nanoindentation revealed that the speleothems with a dominance of calcite were stronger than the speleothems with mixed calcium carbonate polymorphs and silica content. The *in vitro* metabolic activity of the microbial communities associated with the surfaces of the speleothems resulted in calcium carbonate crystal precipitation. Firmicutes and Proteobacteria dominated these populations, in contrast to the populations seen in natural systems. The precipitation of calcium carbonate crystals *in vitro* indicated that microbial metabolic activity may also play an important role in the synthesis and dissociation of biominerals in the natural environment. Our study provides novel evidence of the close relationship between mineralogy, microbial ecology, geochemistry, and nanomechanical properties of natural formations.

## Introduction

Biomineralization is a naturally occurring process where microbial metabolic activity influences the formation of geological structures (Lowenstam, 1981; Hammes and Verstraete, 2002; Hamilton, 2003). A variety of microbial metabolic pathways including photosynthesis, ammonification, denitrification, ureolysis, and methane oxidation have been shown to influence redox conditions and lead to the precipitation of carbonates in different natural environments (Fortin et al., 1997; Douglas and Beveridge, 1998; Zhu and Dittrich, 2016). Bacterial surfaces including cell walls and extracellular polymeric substances (EPS) have also been reported to act as important sites for carbonate mineral nucleation and growth (Douglas and Beveridge, 1998; Bains et al., 2015). The potential for microbes to synthesize carbonates has recently emerged as a prospective technology, called as Microbially Induced Calcium Carbonate Precipitation (MICCP), for civil and environmental engineering applications (Skinner and Jahren, 2004; Phillips et al., 2013; Zhu and Dittrich, 2016). Current usage of the technology includes remediation of heavy metal polluted sites, sequestration of CO_2_, and the construction of biocement (Dhami et al., 2012, 2014, 2016, 2017b; Dejong et al., 2013; Zhu and Dittrich, 2016). Several breakthroughs have been made at laboratory and field scale in the creation and utilization of biomineralized carbonates (De Muynck et al., 2010; Phillips et al., 2013; Dhami et al., 2017a; Porter et al., 2017). The application of this technology offers the advantage of high sustainability due to its synthesis at ambient temperatures and has spurred research into exploration of the properties of naturally mineralized structures (Cacchio et al., 2003; Phillips et al., 2013). Although researchers from specializations such as Geology, Microbiology, Environment, and Engineering have contributed to the information on geological formations from separate perspectives; a connection linking mineralogy, microbiology, and mechanical characterization of such biogenically/abiogenically formed natural formations is lacking. A deeper and fundamental understanding of bio-geo-chemical properties of naturally mineralized structures along with the mechanical properties of these materials is imperative (Northup and Lavoie Diana, 2001; Dhami et al., 2012, 2013b; Zepeda Mendoza et al., 2016).

Geological formations such as caves provide a window into one such environment where mineralized carbonate deposits are formed over geological timescales (Buhmann and Dreybrodt, 1984; Short et al., 2005; Rusznyak et al., 2012). The myriad of rock like deposits in caves are commonly known as speleothems; which vary in range of shapes. These structures are known by different names as stalactites, stalagmites, moonmilk, drip stones, and flowstones depending upon their location (from floors, walls to ceilings) and appearance (soft, spongy, fibrous, or stony) (Borsato et al., 2000; Jones, 2001; Melim et al., 2008). The inorganic and physical chemistry that drive these phenomena is well established (Banks et al., 2010; Pacton et al., 2013), however, a growing body of research indicates that microorganisms can play an important role in carbonate precipitation during speleothem growth (Banks et al., 2010; Rusznyak et al., 2012; Ronholm et al., 2014). Several studies on the analysis of the mineralogy and geochemistry of different cave environments have been conducted (White, 1962; Harmon et al., 1983; Hill and Forti, 1986; Leél-Őssy et al., 2011). The majority of speleothems have been found to be calcareous and composed of calcium carbonate polymorphs such as calcite, vaterite, and aragonite (Ronholm et al., 2014; Demény et al., 2016; Garcia et al., 2016). The growth of speleothems through calcite precipitation has long been viewed as an abiogenic process (Kendall and Broughton, 1978; Broughton, 1983a,b,c). However, mineral deposits that are difficult to explain through geologic or inorganic processes raise the possibility of microbial activity in caves (Jones, 2001). For example, in the case of moonmilk, carbonate precipitation is formed either by direct precipitation of calcite via microbial metabolic activity or by the formation of nucleation surface on which minerals precipitate (Northup et al., 2003; Cuezva et al., 2012). A few studies have also reported the biogenic origins of calcified structures such as “pool fingers” and “U-loops” (Tomczyk-Żak and Zielenkiewicz, 2015). Some studies claim that bacteria produce carbonate minerals as a result of passive growth while others report that chemically reactive cell walls of bacteria act as nucleation sites binding mineral forming elements leading to growth of crystals in oversaturated solutions (Fortin et al., 1997; Barabesi et al., 2007; Dhami et al., 2013a). Researchers have demonstrated that by interacting with minerals, microorganisms play an important role in the formation of caves although little cause and effect has been elucidated (Tomczyk-Żak and Zielenkiewicz, 2015). Cave geochemistry, physico-chemical conditions, and mineralogy has also been reported to have a significant impact on the microbiome and the interactions of these microbes with minerals further play a significant role in the formation and characteristics of biomineralized speleothems (Zepeda Mendoza et al., 2016).

A number of studies have investigated microbial biofilms on cave surfaces (Adetutu et al., 2012; Pacton et al., 2013; Barton et al., 2014; Wu et al., 2015) and others have reported the microbial diversity of caves such as the Lechuguilla Cave in New Mexico (Cunningham et al., 1995; Northup et al., 2003), the sulfidic Frasassi Cave system in Italy, the Movile Cave in Romania (Macalady et al., 2007; Chen et al., 2009), the nitrate/nitrite-dominated Nullarbor Cave in Australia (Holmes et al., 2001), karstic caves of Herrenberg Germany (Rusznyak et al., 2012), and Tjuv-Ante’s Cave in Sweden (Zepeda Mendoza et al., 2016). The existence of varying calcium carbonate polymorphs in these materials has also been related to biogenic activity. A few authors have attributed the precipitation of different types of carbonate deposits in moonmilk to the activity of indigenous microbial populations. These deposits vary from nano-fibers to micro-meter sized needle-fiber crystals in a form of monocrystalline rods and polycrystalline chains (Baskar et al., 2008b; Cuezva et al., 2012; Maciejewska et al., 2017). Microbes have also been reported to mediate a wide range of processes that affect the internal crystal fabric (Jones, 2010) with carbonate precipitation and heterotrophic processes through nitrogen metabolism including ureolysis, ammonification, and nitrate reduction have been reported to play more important roles than photosynthesis (low light conditions) and methanogenesis (Castanier et al., 2000; Banks et al., 2010). Although a few studies have been conducted on the microbial diversity and mineralogy of different cave deposits and speleothems, this area remains relatively unexplored.

Mechanical properties of biomineralized geological formations can provide insights for exploring the potential of microbial carbonate based cements for engineering applications. But in many cases, the non-availability of sufficient quantities of such structures for standard material testing (due to their heritage status) makes it difficult to investigate their mechanical properties. For mechanical testing on (sub) micrometer scale, nanoindentation (also referred to as load and depth-sensing indentation (DSI)) has recently gained attention (Presser et al., 2010). This technique offers the advantage of high accuracy and reliability along with the ability to test small amounts of material; making this method particularly suitable for the speleothems. Fortunately, a few such results are available for rock minerals (Zhu et al., 2007), single calcite crystals (Calvaresi et al., 2013; Dhami et al., 2016), hydroxyapatite crystals (Zamiri and De, 2011), carp otoliths (Ren et al., 2013), and sponge spicules (Müller et al., 2014). This encourages the use of this tool to investigate nano-mechanical properties of different cave speleothems.

Whether the formation of cave speleothems is biogenic or abiogenic or a combination of both is a matter of debate. A few studies have demonstrated that bacteria isolated from cave environments are capable of forming similar crystals from organic calcium salts *in vitro* using acetate rich B4 medium (Rusznyak et al., 2012; Garcia et al., 2016). Maciejewska et al. (2017) found amino acid/peptide ammonification to be more widespread compared to ureolysis in moonmilk formation. The investigation of microbial communities associated with cave speleothems for their biomineralization potential under supplementation of similar organic calcium salts will shed light on the biogenic routes of mineralization, as well as exploring the change in microbial community dynamics under enrichment conditions *in vitro* compared to the native profiles.

This paper aims to characterize the mineralogical, elemental and nano-mechanical properties of different cave speleothems as well as the associated microbiomes. We investigated the biomineralization potential of surface associated microbial communities along with microbial community dynamics under *in vitro* conditions. This is the first study on the collective characterization of mineralogical, microbial, and mechanical properties of cave speleothems to elucidate biogenic mineralization processes.

The Leeuwin-Naturaliste ridge in the Margaret River region of Southwest Western Australia is home to a number of karstic caves stretching for approximately 90 km between Cape Leeuwin and Cape Naturaliste with the Indian Ocean to the west, Geographe Bay to the north and the Southern Ocean to the south (Jasinska, 1997; Eberhard, 2004; Eberhard and Davies, 2011). This provided us with the opportunity to investigate distinctly different cave formations. We examined different cave deposits (stalagmites, stalactites, moonmilk) from three unexplored caves where permission was granted for access. An investigation of mineralized cave speleothems in their natural state as well as under enriched conditions *in vitro* is presented with the aim to: (a) evaluate mineralogical, chemical, and nanomechanical properties of different cave speleothems, (b) elucidate the microbial diversity associated with different cave speleothems and, (c) investigate the potential of speleothem associated microbial communities in the formation of carbonate minerals under *in vitro* conditions as well as identification of microbial communities co-responsible for carbonate precipitation in laboratory conditions for analysis of microbial community shifts in comparison to Natural environments.

## Materials and Methods

### Description of Caves

**FIGURE 1:**
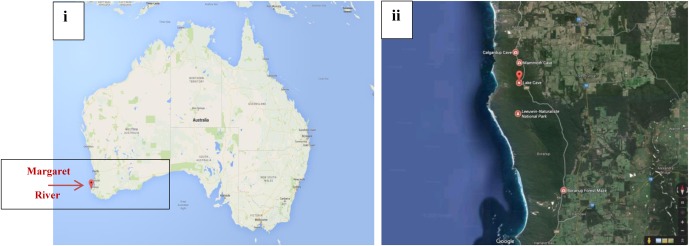
**(i,ii)** Geographical location of Margaret River Caves, Western Australia, Australia [source: Google (Googlemaps, 2016)].

**FIGURE 2:**
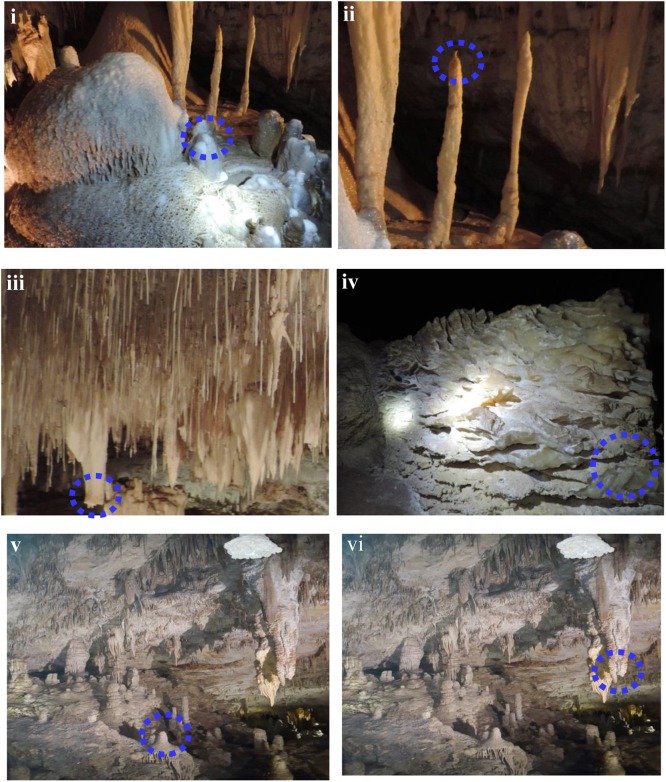
Cave speleothems collected from **(i–iii)** Lake Cave **(iv)** Moondyne Cave **(v,vi)** Mammoth Cave.

Margaret River is located in Western Australia, 277 km (172 mi) SSW of Perth and 48 km (30 mi) SW of Busselton at 33°57′18″S 115°04′30″E. The climate is humid Mediterranean with an average annual rainfall of around 1,130 mm (44 in) (Eberhard, 2004). Complete details about the cave formations and the environment involved have been reported by Eberhard (2004). Permission for the current study was provided by the Augusta Margaret River Tourism Association for sampling calcitic deposits and speleothem sections in a limited number of locations in three caves (Lake Cave, Moondyne Cave, Mammoth Cave) (**Figures 1i,ii**). The speleothems sampled are highlighted in **Figure 2**. In the case of Mammoth Cave, parts of stalactite and stalagmite that had been already shed were provided.

### Sampling of Cave Speleothems

Sampling of calcitic deposits from different formations in each cave (up to 10 g) was conducted using sterile forceps, spatula and chisel. Moonmilk, stalagmite, and stalactite samples were collected from Lake Cave, calcitic deposits were collected from the floor of Moondyne Cave and deposits of stalagmites and stalactites were provided from Mammoth Cave (**Figure 2** and **Table 1**). Sampling locations were selected based on accessibility, regulatory restrictions and special features observed. All the specimens were collected aseptically, transported under refrigeration to the laboratory, and stored at -20°C until use. The samples were separated into three sections in order to correlate mineralogical, mechanical, and microbial characteristics.

**Table 1:** Cave speleothem type, site and labeling.

Site	Speleothem
**Lake Cave**	
1	Moonmilk
2	Stalagmite
3	Stalactite
**Moondyne Cave**	
1	Calcitic floor stalagmite deposit
**Mammoth Cave**	
1	Stalagmite
2	Stalactite

### Determination of Carbon, Nitrogen, and Metal Content

One gram of each sample was added to 10 mL deionized water and shaken briefly. Total Al, Mg, Ca, Fe, Mn, and Na analyses were conducted on samples that were dried at 100°C, extracted with aqua regia (Hoffman, 1991) and analyzed with an inductively coupled plasma mass spectrometer (ICP-MS) (PQ3S; Thermo Electron, United Kingdom) following Rusznyak et al. (2012). For the estimation of Carbon and Nitrogen, the samples were freeze dried, finely ground to 200 mesh size (<75 μm), homogenized and analyzed using a CN analyser (Vario Max; Elementary Analysensysteme GmbH, Germany). Inorganic carbon was determined by measuring the total amount of carbon after removal of organic carbon (OC) and ignition of samples for 4 h at 550°C (Grüneberg et al., 2010). OC concentrations were then calculated from the difference between total and inorganic carbon concentrations. For each test triplicate samples were analyzed.

### Morphological Characterization of Speleothems by SEM

For scanning electron microscopic analysis (SEM) all samples were fixed in 4% formaldehyde in phosphate-buffered saline (PBS) and kept at 4°C until examination. The samples were rinsed in 0.2 M PBS (pH 7.4) for 1 h and dehydrated in a series of graded ethyl alcohol then sputter coated with platinum (thickness of approximately 8 nm) using a SCD005 sputter coater (BAL-TEC, Liechtenstein) to avoid surface charging. Finally, the specimens were investigated with Zeiss Neon 40 EsB dual beam FESEM/FIB-SEM for imaging at 10 kV and WD = 10–11 mm.

### Chemical Characterization of Speleothems by XRD, EDS, and TIMA

X ray diffraction (XRD) spectra were obtained by crushing around 1 g sample of each speleothem and subjecting it to X’ Pert PRO diffractometer with a Cu anode (40 kV and 30 mA). The scanning was done from 3° to 60° 2𝜃 with increments of 0.02° 2𝜃 and a counting time of 10 s per step. The components of the sample were identified by comparing them with standards established by the International Centre for Diffraction Data. For elemental analysis of the samples, the same SEM equipped with Oxford energy dispersive spectrometer (EDS), coupled with INCA 250 system was used to generate elemental maps in the samples. EDS qualitative analysis and elemental mapping was performed at an accelerating voltage of 20 kV. Data acquisition and analysis was done using AZtec software (Oxford instruments, United Kingdom). Five frames, each with an area varying between 0.5 mm^2^ to 1 mm^2^ were mapped. As it can be difficult to identify mineral phases through XRD due to low concentrations or overlapping peaks, poorly crystalline nature of materials with high amorphous content, or high and low temperature variants, the speleothems were also characterized by Tescan Integrated Mineral Analyzer (TIMA). The conditions used for the current analysis were: beam energy 25000 eV, current 6.32 nA, beam intensity 19.56, working distance 15 mm, SEM type TIMA3FE GMU with Pulse Tor 30 detector model at 7.009 μm pixel.

### Mechanical Characterization of Speleothems by Nanoindentation

Cubes of approximately 1 cm were cut from each specimen, embedded in epoxy resin (Buehler eco-thin, Buehler, Lake Bluff, Illinois) and allowed to cure for 24 h (in molds 32 mm × 8 mm). Initial grinding and polishing of samples was performed using silicon carbide paper of reducing gradation 52, 35, 22, and 15 μm to expose the surface. Samples were finally ground and polished using diamond suspensions of reducing gradations as 9, 6, 3, 1, and 0.05 μm on a polishing cloth. Nanoindentation was performed using a G200 nanoindenter (Agilent Technologies) fitted with a Berkovich shaped diamond tip at a Poisson ratio of 0.31. The optical microscope fitted with the nanoindenter was used at 40x magnification to select the points of indentation and a matrix of indentation points on the particles of interest was chosen. At least 50 indentation points were selected for each sample with a minimum spacing between the indents of 10 μm and a maximum depth of indentation set to 1000 nm. The surface approach velocity was set to 20 nm/s and the loading time set to 15 s with a peak hold time of 10 s. On completion, indentation sites were re-inspected with an optical microscope and load-indentation diagrams were plotted. From this data the mechanical properties were estimated (Müller et al., 2014). From the initial slope of the unloading curve, the depth of indentation was estimated. The force corresponding to the displacement was utilized to determine the hardness of the material. The elastic modulus was estimated from the unloading segment of the load–indentation curve. Hardness reflects the resistance of the geological samples to deformation, while the elastic modulus represents the elastic deformation of the material following force application (Müller et al., 2014). A power law function was fitted to the initial unloading portion to determine its slope. The calculations were performed with the software NanoTest Platform Four V.40.08 (Micro Materials Ltd.). We determined Young’s modulus, E, as the maximum slope in the corresponding stress–strain curve. Non-linear behavior at low strains is the result of varying alignment inaccuracy.

### Microbial Characterization of Cave Speleothems

Bacterial cellular activity or Adenosine triphosphate (ATP) activity of the speleothems was analyzed immediately after returning to the laboratory using the BacTiter-Glo Microbial Cell Viability kit (Promega, United States) as described by Barton et al. (2014).

### DNA Extraction from Speleothem Samples

Genomic DNA (gDNA) was extracted in triplicate from all deposits and speleothems. The speleothem sample (1 g) was suspended in sterile phosphate buffer saline and vortexed at high speed followed by sonication in ultrasonic water bath to detach the surface cells. The cell suspension was harvested by microfiltration (0.2 μm pore size filter; Millipore) and the filter washed in PBS. DNA extraction was conducted using the Power Soil DNA kit (MO BIO Laboratories, Carlsbad, CA, United States) following the manufacturer’s instructions. The recovered genomic DNA was pooled and the concentration was quantified using a Nanodrop 8000 Spectrophotometer (Thermo Scientific, Wilmington, DE, United States).

### Amplicon Diversity Sequencing

PCR amplification and sequencing of the V3/V4 region of the 16S rRNA gene was performed by Australian Genome Research Facility (Brisbane, QLD, Australia) using Illumina MiSeq (San Diego, CA, United States) with 2 × 300 base pairs paired-end chemistry. Briefly, extracted gDNA from each of the sample was PCR amplified using primers 341F (5′-CCTAYGGGRBGCASCAG-3′) and 806R (5′-GGACTACNNGGGTATCTAAT-3′). Thermocycling was performed in a Bio-Rad C100 (Bio-Rad Laboratories, Richmond, CA, United States) using AmpliTaq Gold 360 mastermix (Life Technologies, Australia).

### Sequence Analysis

Paired-ends reads were assembled by aligning the forward and reverse reads using PEAR (version 0.9.5) (Zhang et al., 2014). Primers were trimmed using Seqtk (version 1.0)^1^ and the trimmed sequences were processed using Quantitative Insights into Microbial Ecology (QIIME 1.8) (Caporaso et al., 2010) USEARCH (version 7.1.1090) and UPARSE (Edgar et al., 2011) software. Using USEARCH sequences were quality filtered, full length duplicate sequences were removed and sorted by abundance. Singletons or unique reads in the data set were discarded. Sequences were clustered followed by chimera filtering using “rdp_gold” database as the reference. To obtain the number of reads in each Operational Taxonomic Unit (OTU), reads were mapped back to OTUs with a minimum identity of 97%. Using QIIME, taxonomy was assigned using SILVA database (version silva_119) (Quast et al., 2013). The obtained sequences were submitted to National Centre for Biotechnology Information (NCBI) (accession number SAMN07489237 to SAMN07489242 and SAMN07498254 to SAMN07498259). The results are provided as percentage of sequencing reads for the identified OTUs in each sample.

### Enrichment of Speleothem Surface Associated Bacterial Communities and Characterization of Precipitated Carbonate Biominerals

In order to investigate the microbial dynamics and biogenic carbonate mineralization potential of the microbial communities associated with speleothem samples under *in vitro* conditions, one gram of speleothem sample collected from each site was inoculated into flasks containing 100 ml autoclaved B4 medium (4 g yeast extract, 2.5 g calcium acetate, 10 g of glucose) (Banks et al., 2010; Rusznyak et al., 2012). For abiogenic controls, one gram of speleothem samples from each site were subjected to autoclaving in order to remove the associated microbes and then inoculated into sterile B4 media flasks as above. Dry autoclaving may have impacted the mineralogy to but as the aim of this experiment was to differentiate biotic vs. abiotic mineralization, we assumed little effect of the mineral change on crystal precipitations. All the flasks were incubated at 50 rpm in an orbital shaker for 10 days at 30°C in the dark (the very low rpm were to ensure the least loss of surface deposits from the speleothems which may interfere with the precipitates). These cultures were subcultured and grown for another 10 days to monitor the microbial growth (OD_600_) as well as precipitate formation as per Rusznyak et al. (2012). The conditions for *in vitro* carbonate precipitation were significantly different from the actual cave environments with low temperature, high humidity, CO_2_, and minimal nutrients, but the aim of this experiment was to investigate the biomineralization potential of heterotrophic communities associated with speleothems in a shorter time frame. After 10 days, five ml of culture broth was taken from each set, centrifuged at 13,000 ×*g* (4°C) for 10 min and the pellet was harvested for DNA extraction using the Bacterial DNA Isolation Kit (Qiagen^®^) following the manufacturer’s instructions. The microbial diversity of the enriched cultures was analyzed as previously described. For the analysis of the carbonate mineralization potential of the enriched cultures, 10 ml of the culture was filtered using Whatman No. 1 filter paper and the precipitated crystals were harvested as per Zamarreno et al. (2009). The crystals were washed with sterile distilled water, dried at room temperature for 48 h and analyzed for morphological and chemical constituents with SEM and XRD as described previously. All experiments were conducted in triplicate as biological replicates. The data were analyzed by Analysis of Variance (ANOVA) and the means were compared with Tukey’s test. All analyses were performed using Graph Pad Prism software^®^ version 6.0.

## Results and Discussion

### Carbon Nitrogen and Trace Metal Compositions

Carbon/Nitrogen content and major metal concentrations of different cave deposits were analyzed as per Rusznyak et al. (2012) and shown in **Table 2**. The proportion of Ca was very high in all the samples although Si, Mg, Al, S and Fe were also consistently present in varying amounts. Other metals such as K, Na, and P were recorded in small quantities. The calcium content was lower in the Mammoth Cave speleothems than those of the other two caves. This was also seen for the calcium proportion as carbonate with Lake Cave and Moondyne Cave speleothems having a higher content compared to Mammoth Cave speleothems. Interestingly, the Mammoth Cave samples were the only ones with detectable levels of Silica. Mineral dissolution was observed in the speleothems of Mammoth Cave (shown in the SEM images in next section). The high amount of silica in this case may be an indicative of sandy sediments being washed into the cave or be due to the dissolution of the speleothem or speleogenesis. Previous studies have reported the role of microbial activity in speleogenesis (Banks et al., 2010). Notably the presence of Mg, S, Al and Fe were also higher in Mammoth Cave speleothems suggesting different formation mechanisms and siliceous mineralogy in these samples. A number of cave speleothems have been reported to have similar calcium contents due to the calcitic nature of these formations but variations in the distribution of other metals and elements in the different samples of the study may influence the microbiomes of the speleothems as well as their mechanical properties (Melim et al., 2001; Baskar et al., 2008a; Cacchio et al., 2012; Rusznyak et al., 2012; Banerjee and Joshi, 2014).

**Table 2:** Carbon, Nitrogen, CaCO_3_ and trace metal content of different cave speleothems^∗∗^.

Sample	C (%)	N (%)	Ca as CaCO_3_ (%)	Ca (mg/kg)	Si (mg/kg)	K (mg/kg)	Mg (mg/kg)	Na (mg/kg)	S (mg/kg)	Al (mg/kg)	Fe (mg/kg)	P (mg/kg)
**Lake Cave**											
Moonmilk	4.2 ± 1.3	0.01	99 ± 0.1	408000 ± 1500	<100	<100	924 ± 23	273 ± 5	1360 ± 43	138 ± 27	53 ± 18	115 ± 21
Stalagmite	4.8 ± 1.2	0.01	99 ± 0.2	403000 ± 2100	<100	<100	1160 ± 87	136 ± 18	1290 ± 36	376 ± 29	298 ± 46	62 ± 11
Stalactite	4.9 ± 1.7	0.02	93 ± 3.6	392000 ± 2700	<100	<100	1470 ± 65	437 ± 29	1310 ± 68	1390 ± 76	655 ± 54	859 ± 42
**Moondyne Cave**											
Stalagmite	4.8 ± 1.8	0.02	94 ± 2.3	391000 ± 1900	<100	<100	1380 ± 84	415 ± 16	1270 ± 37	1310 ± 82	620 ± 42	825 ± 52
**Mammoth Cave**											
Stalagmite	5.4 ± 1.5	0.01	77 ± 4.9	321000 ± 1600	180 ± 24	<100	3190 ± 76	597 ± 22	1710 ± 122	2640 ± 53	1080 ± 85	145 ± 28
Stalactite	5.3 ± 1.3	0.02	75 ± 3.8	320000 ± 2900	198 ± 32	<100	3380 ± 88	458 ± 31	1660 ± 88	2790 ± 48	1280 ± 94	125 ± 19

*^∗∗^Values are mean ± SD (*n* = 3).*

### Morphological, Mineralogical and Mechanical Properties

**FIGURE 3:**
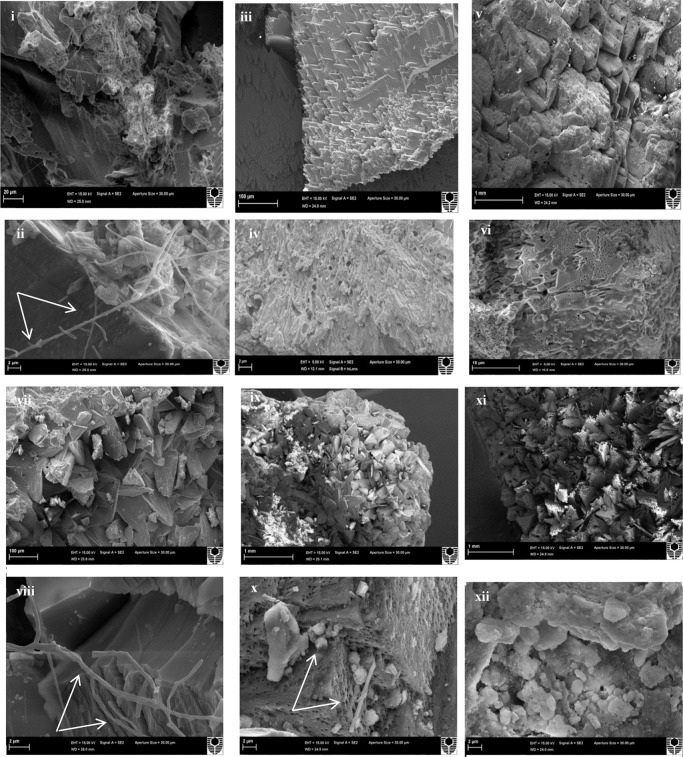
Scanning electron micrographs of: **(i,ii)** Lake Cave moonmilk; **(iii,iv)** Lake Cave stalagmite; **(v,vi)** Lake Cave stalactite; **(vii,viii)** Moondyne Cave stalagmite; **(ix,x)** Mammoth Cave stalagmite and **(xi,xii)** Mammoth Cave stalactite (Arrows indicate hyphal networks and microborings) at lower and higher magnifications.

**FIGURE 4:**
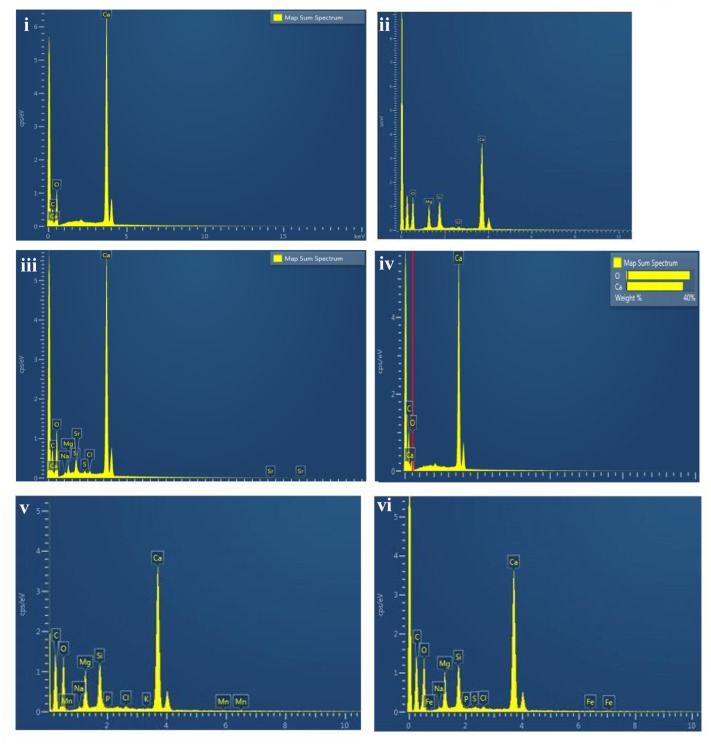
Energy dispersive X ray spectrum of: **(i)** Lake Cave moonmilk; **(ii)** Lake Cave stalagmite; **(iii)** Lake Cave Stalactite; **(iv)** Moondyne Cave stalagmite; **(v)** Mammoth Cave stalagmite and **(vi)** Mammoth Cave stalactite.

**FIGURE 5:**
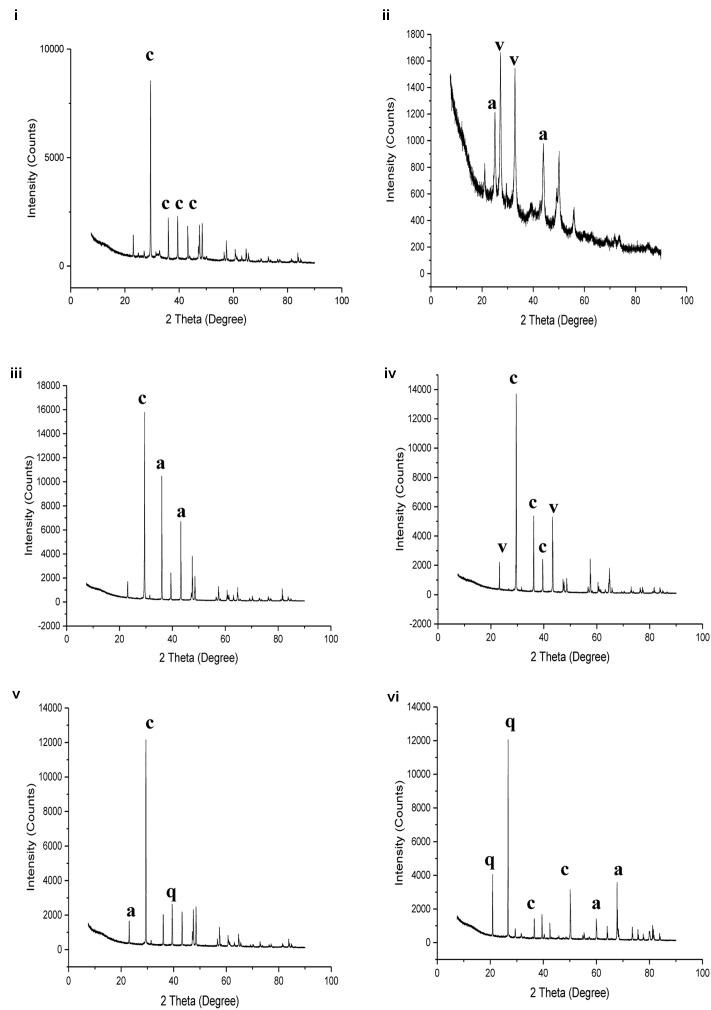
X ray diffraction analysis of: **(i)** Lake Cave moonmilk; **(ii)** Lake Cave stalagmite; **(iii)** Lake Cave Stalactite; **(iv)** Moondyne Cave stalagmite; **(v)** Mammoth Cave stalagmite and **(vi)** Mammoth Cave stalactite (where c denotes calcite; a denotes aragonite; v denotes vaterite; q denotes quartz).

**FIGURE 6:**
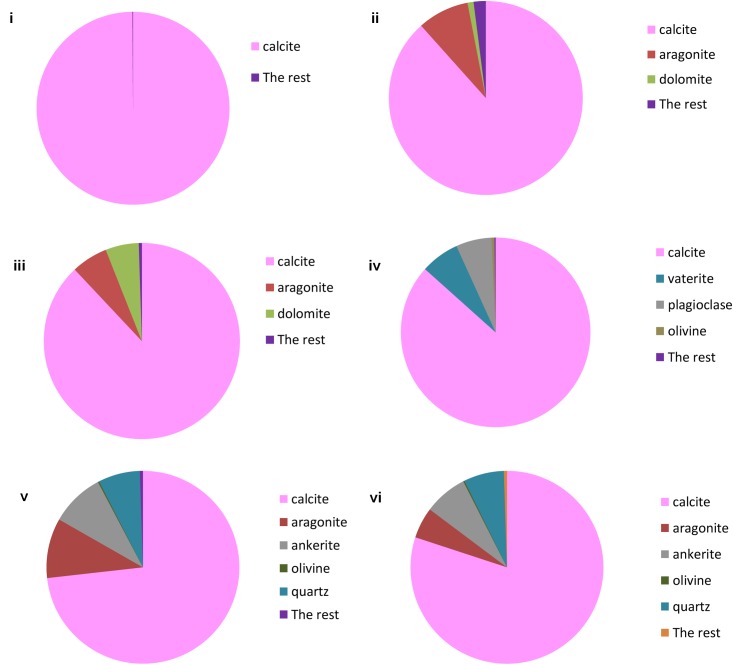
TIMA analysis of: **(i)** Lake Cave moonmilk; **(ii)** Lake Cave stalagmite; **(iii)** Lake Cave Stalactite; **(iv)** Moondyne Cave stalagmite; **(v)** Mammoth Cave stalagmite and **(vi)** Mammoth Cave stalactite.

**FIGURE 7:**
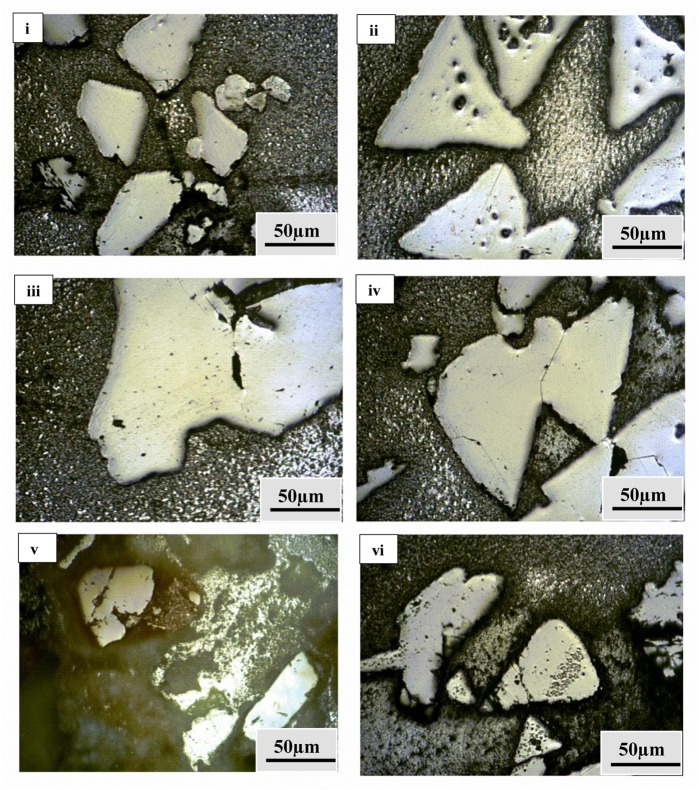
Microstructures for nanoindentation through optical microscope fitted with Nanoindenter for: **(i)** Lake Cave moonmilk; **(ii)** Lake Cave stalagmite; **(iii)** Lake Cave Stalactite; **(iv)** Moondyne Cave stalagmite; **(v)** Mammoth Cave stalagmite and **(vi)** Mammoth Cave stalactite.

Scanning electron microscopy observations of the speleothems showed a variety of crystalline mineral formations together with a variety of microbial morphotypes and mineralized cells/filaments (**Figure 3**). While there were morphological differences between the different speleothems, most of them displayed associations with hyphae or biofilms. Elemental analysis by EDS, mineralogical analysis by XRD and TIMA demonstrated the presence of multiple mineral polymorphs in the speleothem samples (**Figures 4**–**6**). The microstructures of the speleothem sections for indentation can be seen in **Figure 7**. The elastic modulus and hardness was determined from the load indentation graphs and the values are presented in **Table 3**.

**Table 3:** Mechanical properties of different cave speleothems^∗∗^.

Sample	Elastic Modulus at Max Load (GPa)	Hardness at Max Load (GPa)
Resin	6 ± 0.8	0.08 ± 0.02
Quartz (Zhu et al., 2007)	105 ± 5	13 ± 1
Feldspar (Zhu et al., 2007)	85 ± 5	9 ± 1
Calcite (Ren et al., 2013)	72 ± 11	1.98 ± 0.31
Vaterite (Ren et al., 2013)	39.13 ± 8.04	1.38 ± 0.39
Lake Cave moonmilk	126.4 ± 6.2	17.10 ± 3.2
Lake Cave stalagmite	80.5 ± 3.9	12.86 ± 2.1
Lake Cave stalactite	77.5 ± 5.4	6.2 ± 1.5
Moondyne Cave stalagmite	78.7 ± 3.68	9.1 ± 1.15
Mammoth Cave stalagmite	69.13 ± 5.5	3.5 ± 1.41
Mammoth Cave stalactite	64.39 ± 1.95	3.12 ± 0.8

*^∗∗^Values are mean ± SD (*n* = 10).*

The moonmilk sample was characterized by a mixture of thickly laminated columnar crystals which were widely distributed (**Figures 3i,ii**). The contacts between different layers were associated with rhombohedral crystals and a few cavities were also noticed. The size of the crystals generally varied from 20 to 200 μm but some larger crystals were recorded. A few regions appeared to be covered by a filamentous network of hyphae and cells. The elemental and mineralogical analysis revealed that the sample consisted entirely of calcite and was composed of Ca, C, and O (**Figures 4i**, **5i**, **6i**). The organic composition appeared to be high as analyzed from a high Carbon peak in the elemental analysis. XRD and TIMA both demonstrated the presence of pure calcite as the mineral phase. Similar structures were also reported in moonmilk deposits of the Ballynamintra karstic Cave (Rooney et al., 2010). The precipitation of calcite moonmilk is generally attributed to inorganic processes but a few studies have suggested the formation of it through both biogenic and abiogenic processes (Onac and Ghergari, 1993; Hill and Forti, 1997).

The stalagmite sample from Lake Cave had an abundance of triangular crystals interconnected by smaller cementing binders (**Figures 3iii,iv**). All the components seemed radially arranged. Jones (2001) reported similar structures to be formed in cave deposits due to destructive processes of etching and boring. The formation of spiky calcite crystals was reported to be influenced by microbial activity and mucus leading to irregular shaped depressions (Folk et al., 1985). A closer view revealed encrustations of some bacterial cell like fossilized imprints. EDS analysis revealed the presence of Ca, C, and O along with small peaks of Si and Na (**Figure 4ii**). The carbonates were identified as calcite by XRD while TIMA also confirmed the presence of aragonite (**Figures 5ii**, **6ii**).

The stalactite sample of Lake Cave was characterized by alteration of thickly laminated columnar and accretionary crystals in the size range of 500 μm to 1mm which were widely distributed (**Figures 3v,vi**). In some areas the crystals seemed to be compactly cemented. A cover of matrix like film was also recorded in a few sections with some bacterial like imprints. The current sample displayed microbial like associations on the surface, but whether they are playing an associative or dissociative role is not certain. The hypothesis that the growth of stalagmites and stalactites is mediated by microbial activity is postulated in many studies but there are several challenges to identify these processes in natural systems due to fossilization (Barton et al., 2007; Melim et al., 2008). Elemental analysis again revealed the presence of high C, Ca, O, and Mg along with small peaks of Cl and Na (**Figure 4iii**). Unusual mineral morphologies coupled with high Mg content have also been related to EPS influenced mineralization as biofilms help initiate layer formation on the stalactites via organomineralization processes (Banks et al., 2010; Adetutu et al., 2012). The mineralogical analysis demonstrated the presence of calcite and aragonite though XRD along with dolomite identified through TIMA (**Figures 5ii**, **6iii**).

The stalagmite sample from the Moondyne Cave displayed a slightly different morphology with sharp triangular prismatic crystals with acicular needles in a radial arrangement (**Figures 3vii,viii**). The size of the crystals varied from 20 μm to 100 μm with the bigger crystals cemented by smaller crystals. In this case, the rhomboids had regular outlines with possible microbial hyphae over them. Pacton et al. (2013) reported the presence of cement filled microborings in stalactites of the Botovskaya Caves. Mineralogical analysis of the speleothems in Moondyne Cave revealed that they are composed of calcium carbonates in the form of calcite and vaterite along with a feldspar mineral plagioclase (**Figures 4iv**, **5iv**, **6iv**).

The speleothems collected from Mammoth Cave had different morphological features to those from the other caves due to variety of diagenetic processes, climate and associated environments (Demény et al., 2016). The stalactite sample displayed sharper trigonal prismatic crystals (**Figures 3ix,x**). The empty microborings were clearly seen on the surface of calcite crystals that were open and flaky. Pacton et al. (2013) in their study found that microborings were generally composed of high Fe-Si phases and Mg-calcite precipitates which are corroded by iron oxides. The microborings were seen associated with EPS in their study. In this case also, partial dissolution of the rhombohedral crystals was observed in some sections. The previous elemental analysis of these samples displayed high content of Si and Fe along with Ca, C, and O (**Figure 4v**) paving way to the hypothesis that iron oxides might be responsible for corroding these sections and hinting the role of EPS. XRD and TIMA revealed the crystal phases as a mixture of calcite and aragonite along with quartz and ankerite (**Figures 5v**, **6v**). Ankerite is closely related to dolomite differing in the presence of iron and manganese instead of magnesium. This mineral has also been found in the Jenolan Caves in New South Wales, Australia (Pogson et al., 2014).

Another speleothem from the Mammoth Cave had rosette like crystals surrounded by flaky aggregates (**Figures 3xi,xii**). The contacts between adjacent crystals appeared to be filled by cement like binders with EPS encrusted mineral precipitates. Some areas revealed a breakdown of the substrate which could be related to the etching/boring activity of microbes (Jones, 2001). Some crusts showed corroded features. Similar features were displayed in stalactites of the Botovskaya Cave (Pacton et al., 2013). EDS analysis in this case also showed the presence of high amounts of Si together with Ca, C, O, Mg, and Fe while the mineralogical analysis demonstrated the presence of calcite, aragonite, dolomite, and quartz (**Figures 4vi**, **5vi**, **6vi**). The presence of intermediate forms and higher amounts of Mg in the Mammoth Cave may be an indicator of active mineralization. The mineralogical and micrographical analysis was closely related to the metal composition.

The variety of textures, morphology, and mineralogy of the different speleothems indicates different processes and controls in the formation of these structures. Different mineralogies and polymorphs such as calcite, vaterite, and aragonite have been analyzed by several studies (Sánchez-Román et al., 2007; Pacton et al., 2013; Demény et al., 2016). Self and Hill (2003) reported that the precipitation of calcite causes enrichment of Mg which favors the formation of aragonite and in a few cases leads to the precipitation of hydro magnesite. In another study, Morse (1983) and Sánchez-Román et al. (2007) explained differences in the mineralogies by kinetic analysis. It was reported that aragonite and calcite nucleate and grow at the same time, but in different settings within the cave. A more continuous water supply in pools and some stalactites promote calcite nucleation, whereas seepage and drip water provided a more favorable environment for aragonite, which forms more delicate speleothems. The variations of mineralogies may also be indicative of different stages of speleothem formation and a varying geochemistry. A recent study of cave speleothems by Demény et al. (2016) reported the transformation of amorphous calcium carbonate into calcite in the presence of cave drip waters where aragonite and vaterite were seen to be intermediate forms, whose formation is dependent upon pH and the concentration of Mg.

**FIGURE 8:**
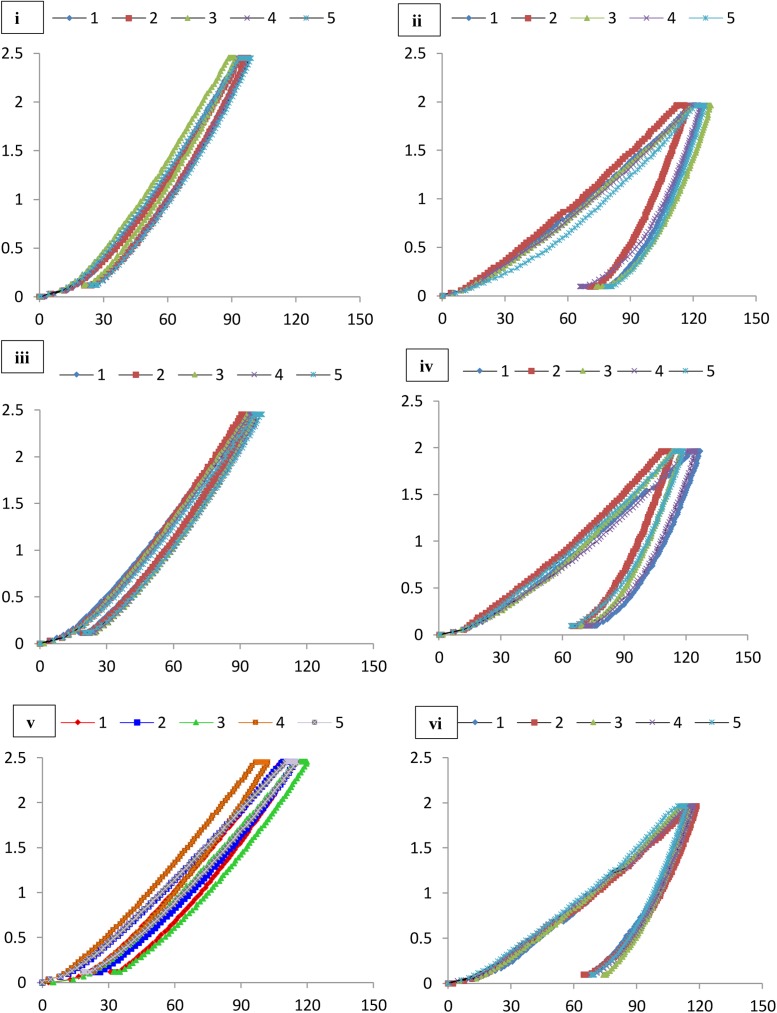
Load indentation graphs of **(i)** Lake Cave moonmilk; **(ii)** Lake Cave stalagmite; **(iii)** Lake Cave Stalactite; **(iv)** Moondyne Cave stalagmite; **(v)** Mammoth Cave stalagmite and **(vi)** Mammoth Cave stalactite.

Nanoindentation was used to study the microscale mechanical properties of these formations (**Figures 7**, **8** and **Table 3**). This tool also helps in the analysis of the different phases of a material together with the properties of the interfacial regions. Maintenance of sterile conditions is difficult as the methodology depends upon casting of the materials within resins. Therefore we aimed to determine the overall mechanical properties of the different speleothem samples and investigate the relationship to mineralogy rather than the effect of microbial interactions on the mineral surface. Further tests to determine microbial-mineral interactions and their effect on the nanomechanical properties in the original samples can be conducted using Atomic Force Microscopic techniques.

The mineral constituents of the exposed surface of the speleothem samples after polishing were carefully indented without the interference of the resin matrix (Dhami et al., 2016). The typical microstructures of different speleothem samples recorded through an optical microscope fitted with the Nanoindenter are shown in **Figure 7**. The sites for indentation were selected to cover at least 50% of the image area. Any analysis points on the resin or the interphase between the resin and the sample were discarded from the final analysis. The load indentation curves of the samples are presented in **Figure 8**. Significant variations were recorded between the different speleothems which in some cases were related to their mineralogy. It was observed that the elastic modulus of the speleothems varied within a range of 76–126 GPa while the hardness varied from 2.20 to 20.10 GPa. The Lake Cave Moonmilk had a hardness value of 14–20 GPa and modulus in the range of 120–132 GPa while the stalagmite sample had a hardness value of 10–14.4 GPa with an elastic modulus of 77–84 GPa (**Figures 8i,ii** and **Table 3**). In the case of the Lake Cave stalactite, the hardness and modulus values varied from 5.3–7.5 GPa to 72–83 GPa (**Figure 8iii**). The variation in the mechanical properties was smaller for the stalagmite sample compared to the stalactite sample. It was noticed that the Moonmilk specimen had the highest strength properties which may be in relation to its higher calcite content. In both of the other speleothems, other polymorphs of calcium carbonate were also recorded along with calcite which may play a role in determining the properties of these mineralized formations. However, further investigations of different sections of the same speleothem would be necessary to obtain a more accurate picture. The Moondyne Cave stalagmite had a slightly lower hardness property compared to the Lake Cave samples with a hardness values of 8–10.2 GPa and an elastic modulus value of 75–82 GPa (**Figure 8iv** and **Table 3**). In this case, there was also considerable variation across the cross section of the specimen. The previous mineralogical analysis of this sample had revealed the presence of mixed crystals (small and large) composed of different mineral phases such as calcite, vaterite, and plagioclase and these may be responsible for variations observed. The Mammoth Cave speleothems had the lowest mechanical properties (**Figures 8v,vi**). The stalactite demonstrated hardness in the range of 2.15–4.9 GPa and a modulus value of 64–74 GPa compared to the stalactite sample with hardness and modulus values varying from 2.3-3.9 GPa to 62–66 GPa. Both of these deposits had a much higher content of silicate mineral along with Mg–Fe, and there were also signs of dissolution which may have affected their pore fraction (**Figures 3ix,x**, **4v,vi**). These features may have been responsible for the lower mechanical properties compared to the calcite rich mineralized deposits.

It is believed that different crystal orientations are responsible for differences in mechanical properties (Alonso-Zarza and Wright, 2010). Even within the same sample, there appeared considerable variation in the properties which may be related to the age of the variable accretions inside the geological structures but further studies are required to investigate this hypothesis. There have only been a few reports on the nanomechanical properties of multiphase materials (Constantinides and Ulm, 2007; DeJong and Ulm, 2007; Zhu et al., 2007). Zhu et al. (2007) found that for quartz, the modulus values were 100–110 GPa and the hardness values were 12–14 GPa while orthoclase feldspar recorded modulus values of 80–90 GPa and hardness values of 8–10 GPa. They reported that different crystal orientations of isotropic minerals contribute to the differences in their mechanical properties. The calcium atoms in calcite have a sixfold coordination, nine-fold in aragonite and eightfold coordination in vaterite playing a role in their mechanical properties (Wang and Becker, 2009). It has also been found that the position of CO_3_^2-^ ions in different polymorphs such as vaterite are uncertain (disorder displacement); where Ca^2+^ and CO_3_^2-^ are not as closely packed as in calcite and aragonite crystals and therefore their mechanical properties are variable (Ren et al., 2013). Müller et al. (2014) found that the elastic modulus of pure calcite prisms is 72.83 ± 11.68 GPa in comparison to vaterite deposits at 39.13 ± 8.04 GPa but as geological formations are mixtures of different minerals and elements, their final properties vary based upon the crystal orientations and growth stages. This study is the first where an attempt to investigate the mechanical properties of mineralized natural formations was made, however, a comparison of nanoindentation with bulk property testing is essential for a full understanding.

Along with the physical, climatic, environmental, geographical, and chemical factors that play a role in speleothem formation, a few studies have found that microbes can also influence the growth (and dissolution) of these structures by microbially mediated mineralization through trapping and binding of detrital grains on the substrate, and/or by mediating mineral precipitation (Cañveras and Sanchez-Moral, 2001; Jones, 2001; Baskar et al., 2008a; Banks et al., 2010; Pacton et al., 2013). It is difficult to investigate the role of microbes or demonstrate a cause and effect relationship at this stage, but the study of their associations and diversities may shed more light on biogenic and abiogenic processes in naturally mineralized structures.

### Microbial Characterization of Cave Speleothems

In order to test for the presence of active microbes associated with the speleothems, a luminescence based ATP test was conducted. Relative luminescent units (RLU) varied from 240 in the Lake Cave stalagmite to 2320 RLUs in the Moondyne Cave stalagmites which signified high microbial activity. Similar microbial activity was seen In the case of speleothems of Venezuelan Cave (Barton et al., 2014). While the detection of microbial activity is not necessarily an indicator of microbial mineralization/dissolution processes in the speleothems, it does confirm their presence in these oligotrophic environments.

**FIGURE 9:**
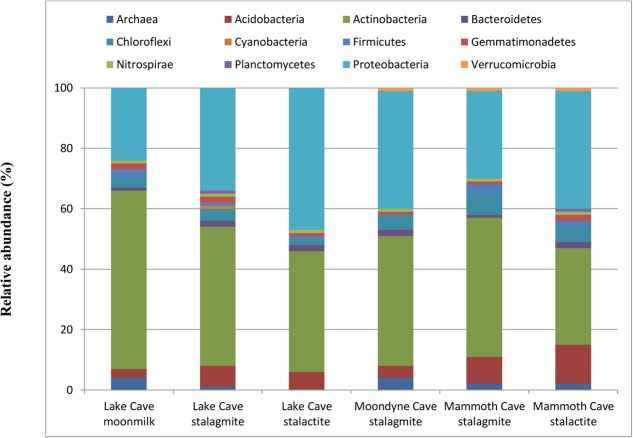
Bacterial population composition at Phylum level associated with different Cave speleothems (based upon 16S rRNA gene sequencing presented as a fraction from total population).

The diversity of microbial communities from the speleothems was analyzed. The sequences were grouped into OTUs and classified using the GreenGene database. Information regarding OTUs and sequencing reads is available in the Supplementary Section. Large variation in microbial community structure associated with the different cave speleothems was observed at phylum level (Family and genus level in Supplementary Data) (**Figure 9**).

The microbial community structure of the Lake Cave speleothems revealed huge richness and variability in OTUs. The Moonmilk sample from Lake Cave was associated mainly with Actinobacteria (59%), Proteobacteria (24%) along with Firmicutes (2%). In contrast, the stalagmite sample was dominated by Actinobacteria (46%) and Proteobacteria (34.2%). In the case of the stalactite sample from this cave, again there was a prevalence of Proteobacteria (47%) and Actinobacteria (40%). The Lake Cave stalactite had a relative abundance of both α and β Proteobacteria (*Sphingomonadaceae* and *Oxalobacteraceae*) at family level (Supplementary Material) while the Lake Cave stalagmite sample had a higher prevalence of *Streptomycetaceae* as was also seen by Maciejewska et al. (2017). The stalagmite from Moondyne Cave was dominated by Actinobacteria (43%) and Proteobacteria (39%) along with Chloroflexi (5%). In the case of the Mammoth Cave speleothems, noticeable variations were observed compared to the other speleothems. At phylum level Actinobacteria (46%) dominated the stalagmite sample followed by Proteobacteria (29%) and Acidobacteria (9.2%). In the stalactite, Proteobacteria were more abundant (39%) followed by Actinobacteria (32.3%) with a higher level of Acidobacteria (13%). The Mammoth Cave speleothems had a much higher proportion of Acidobacteria compared to the other speleothems. At family level, an abundance of Solirubrobacterales and Rhizobiales from the Actinobacteria and α Proteobacteria was observed.

The major phyla in all speleothems were Proteobacteria, Actinobacteria, and Acidobacteria. In general, the dominance of Proteobacteria has been related to their chemoorganotrophic/chemolithotrophic nature. Success of Proteobacteria colonization in several cave environments may be attributed, in part, to their ability to degrade a wide range of organic compounds, their ability to fix atmospheric carbon and transform nitrogen (Tomczyk-Żak and Zielenkiewicz, 2015). An abundance of Proteobacteria (Alpha, Beta, Delta, and Gammaproteobacteria subclass) has also reported in the karstic caves of Lascaux, Tito Bustillo Caves, Altamira Caves, and Herrenberg Cave Germany (Portillo et al., 2008; Bastian et al., 2009; Rusznyak et al., 2012). The dominance of Sphingomonodales and Rhizobiales in speleothems from Lake and Moondyne Caves support high nitrogen fixation abilities in such oligotrophic environments (Larouche et al., 2012). The presence of a large number of heterotrophic, nitrogen fixing species suggests that microorganisms depend on surface derived carbon for growth, while their ability to fix nitrogen plays a critical role in their survival. Actinobacteria have been reported to play an active role in carbonate biomineralization. Their prevalence was highest in the moonmilk sample from Lake Cave that was composed of pure calcite. Although the formation of moonmilk through biogenic or abiogenic process is controversial, and the dominance of biomineralizing Actinobacteria does not indicate whether these communities subsided on these minerals either during or after their formation, there is a close relationship between the mineralogy and the type of microbial structures. The dominance of Streptomyces in high calcium carbonate rich speleothems was also noted which agrees with previous reports of carbonate mineralization potential of such microbes (Maciejewska et al., 2017). In the case of the Altamira Caves, Streptomyces played a major role in capturing CO_2_ and precipitating calcium carbonates under low humidity/ CO_2_ conditions (Cuezva et al., 2012)_._ Firmicutes (*Bacillus*) have been widely found to be involved in calcite precipitation (Zepeda Mendoza et al., 2016) and were quite noticeable in the Lake and Moondyne Cave calcitic speleothems. At genus level, a large number of previously reported carbonate precipitating bacteria associated with cave speleothems of Lake and Moondyne Cave were observed including species of *Sporosarcina, Bacillus, Lysinibacillus, Exiguobacterium, Myxococcus, Paenibacillus, Geobacillus, Syneccococcus, Arthrobacter, Nitrospira, Pseudomonas* (Rusznyak et al., 2012; Wu et al., 2015; Garcia et al., 2016). The phylum Acidobacteria, which was found to be more dominating in Mammoth Cave speleothems, has previously been reported to play an active role in sulfur and iron reduction (Baskar et al., 2008a). The Mammoth Cave speleothems had a lower carbonate content, high Fe and S content as well as surface borings which indicate that these microbes may be playing an active role on the surface of such substrates. Wu et al. (2015) emphasized the impact of cave habitat types such as wall deposits, soils, and aquatic sediments on the bacterial community composition. Previous studies have postulated that the growth of stalactites and stalagmites is mediated by microbes (Cunningham et al., 1995; Jones, 2001; Melim et al., 2001; Baskar et al., 2008b). The diversity studies have confirmed the presence of specific classes of bacteria associated with the speleothems in close connection to their mineralogical composition, however, it does not inform us of the role of microbial formative or dissociative during growth and development. Although mineralogy seems to be the driving force behind the microbial community composition in this study, affecting the properties of these structures, further studies of active cave formations using recent OMICS tools should be conducted to gain further knowledge of the influence of microbes in real environments.

### Enrichment of Mineralizing Communities and Characterization of Biominerals

Microbial carbonate precipitation processes in Cave environments have been reported to be primarily driven by heterotrophic communities which alter the local conditions to promote CaCO_3_ precipitation (Castanier et al., 2000; Banks et al., 2010; Rusznyak et al., 2012; Maciejewska et al., 2017). In order to gain insights into the actual biogenic process associated with mineral precipitation in caves, mimicking cave conditions in the laboratory will be imperative, including maintenance of similar temperature cycles, humidity, dark conditions, minimal nutrients, and subsurface situations. Establishing field-like conditions in the laboratory has always been challenging, so most of the previous studies investigating the role of biogenic carbonate formation have relied upon the use of acetate rich B4 media at ambient temperatures (Zamarreno et al., 2009; Banks et al., 2010; Shirakawa et al., 2011; Rodriguez-Navarro et al., 2012). Although rich nutrient conditions and ambient temperatures in the laboratory are not a true representation of actual cave processes, such experiments do shed light on the carbonate biomineralization potential of native microbial communities.

**FIGURE 10:**
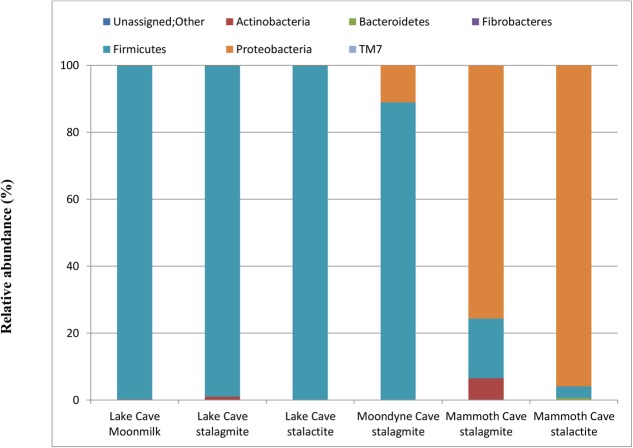
Bacterial population composition at Phylum level in laboratory enriched conditions (based upon 16S rRNA gene sequencing presented as a fraction from total population).

In order to further confirm the presence and association of speleothem surface microbes, their growth in B4 media was recorded. A significant increase in optical density after 4 days was recorded in all the biogenic sets (varying from 1.2 ± 0.31 – 2.6 ± 0.53) while little change was observed in the abiogenic set (with sterile speleothem sample input). The community structure and mineralization potential of the enriched cultures were then investigated. The relative abundance at Phylum level of the enriched communities under *in vitro* conditions can be seen in **Figure 10** and the relative abundance at genus level of the dominant microbial communities in all treatments is presented in Supplementary Table 2.

Variation in the composition of the enrichment microbial community were observed but Firmicutes were found to be the dominant phylum for Lake Cave and Moondyne Cave cultures while Proteobacteria were dominant in Mammoth Cave enrichments. Firmicutes and Proteobacteria has been previously found to be common genera involved in biomineralization (Rusznyak et al., 2012; Wu et al., 2015; Garcia et al., 2016; Andrei et al., 2017). Although Proteobacteria and Actinobacteria were dominant in the natural cave speleothems, under B4 media enrichments, Firmicutes were found to be the most prevalent along with Proteobacteria.

In the case of the Lake Cave enrichments, Firmicutes completely dominated the culture with a relative abundance of 99% while in the Moondyne Cave speleothem enrichments, it reached around 88% from an initial composition of around 1%. In the case of Mammoth Cave speleothems, dominance of Proteobacteria was recorded. The Firmicutes in case of Mammoth Cave were recorded at 17% in the stalagmite enrichments and around 3% in case of stalactite sample. At genus level, the Lake Cave moonmilk and stalactite enrichments comprised mainly of *Bacillus* while the stalagmite enrichment had a prevalence of *Planococcus* (Supplementary Table 2). In the case of the Moondyne speleothem sample, *Bacillus* and *Brevibacillus* genus dominated. The majority of communities in Mammoth Cave enrichment were found to be gram negative; belonging to *Caulobacter* and *Burkholderiales* genera in case of stalagmite sample and *Pseudomonas* in stalactite enrichments.

Firmicutes belonging to the Bacilli family have previously been reported to be the most dominant phylum in laboratory studies on bacteria isolated from cave environments (Cacchio et al., 2003; Banerjee and Joshi, 2014; Garcia et al., 2016). Cacchio et al. (2003) found that *Bacillus* sp. represented about 63% of all isolates from a limestone cave and a loamy soil. Recently Garcia et al. (2016) found that *Bacillus* sp. dominated the genera isolated from saline soils. Strains of *Pseudomonas* have also been isolated in a few studies from speleothems in caves (Chekroun et al., 2004; Rusznyak et al., 2012; Garcia et al., 2016). In a recent study of Andrei et al. (2017), both *Bacillus* and *Pseudomonas* genera were found to be the predominant strains enriched from a limestone statue. It was also demonstrated that the predominance of *Pseudomonas* is linked to its common occurrence in soils. In this study, more *Pseudomonas* was enriched from the silica rich Mammoth Cave speleothems compared to calcitic speleothems suggesting a connection. The increased biomass (in biogenic sets) and observed diversity indicate the potential of acetate rich B4 media in promoting the growth of culturable bacteria from cave speleothems under laboratory conditions. However, it also highlights the shift in community structure seen in laboratory conditions which proves that nutrient availability and physical conditions play crucial role in selection of microbial metabolism and community structure. As laboratory enriched community structures are completely different from the natural conditions, preferring a few communities over the others; this study highlights the importance of future studies wherein natural conditions should be mimicked more closely. Variations in microbial community structures associated with different speleothems grown in different media have also been reported by other researchers (Rusznyak et al., 2012; Garcia et al., 2016; Maciejewska et al., 2017). Continuous monitoring of community shifts over regular intervals of time under those conditions will provide a better picture of the real field mechanisms.

**FIGURE 11:**
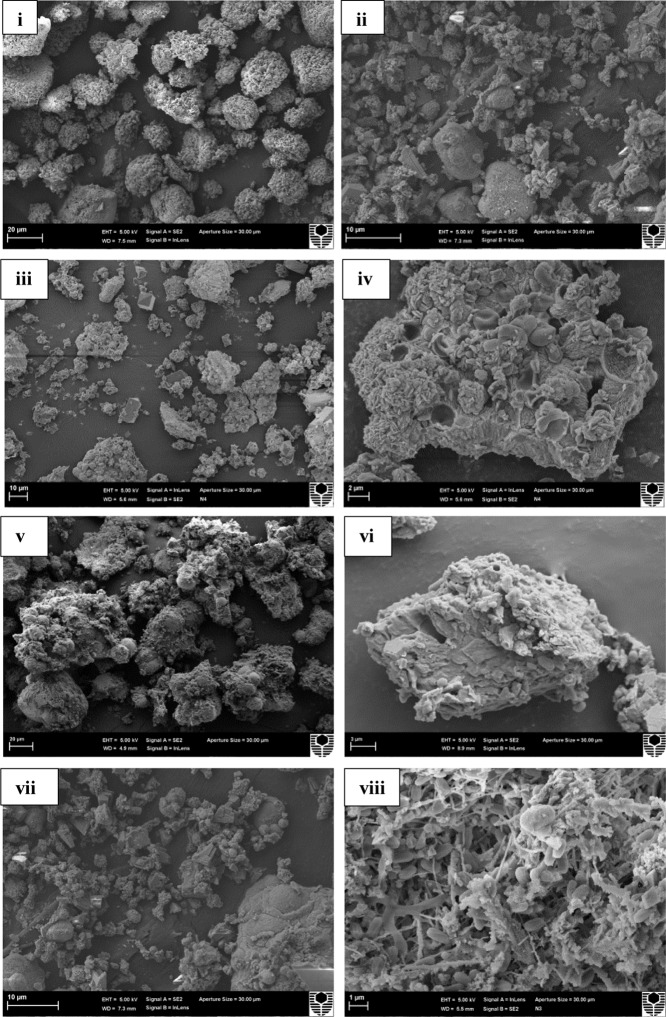
Scanning electron micrograph of crystals precipitated in **(i,ii)** Lake Cave moonmilk enrichment set; **(iii,iv)** Lake Cave stalagmite enrichment set; **(v,vi)** Moondyne Cave stalagmite enrichment set **(vii,viii)** Mammoth Cave stalagmite enrichment set.

**FIGURE 12:**
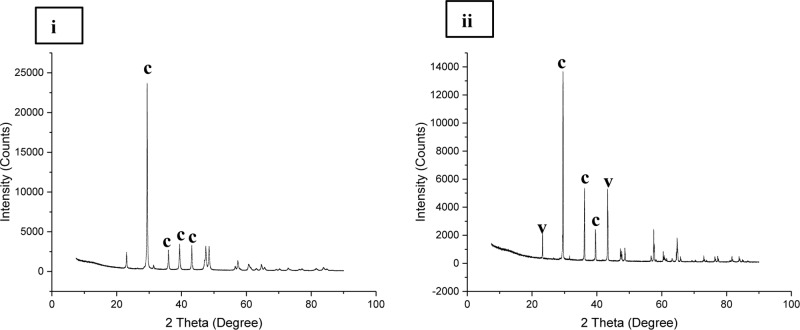
X ray diffraction analysis of carbonate crystals precipitated in **(i)** Lake Cave moonmilk enrichment set **(ii)** Mammoth Cave stalagmite enrichment set (where c denotes calcite; v denotes vaterite).

Micrographic and mineralogical analysis of the crystals precipitated by microbes associated with the speleothems grown in B4 medium showed successful precipitation in all the biogenic speleothems. SEM analysis of the crystals confirmed the formation of crystals of varying sizes in all the biotic sets. **Figure 11** shows crystals formed by bacterial communities associated with different speleothems under laboratory conditions. In general, a mixture of smooth, rhombohedral, and spherical crystals with sizes varying from 20 to 100 μm was observed in the case of enrichments from Lake Cave (**Figures 11i–iv**), rhombohedral crystals in the case of enrichments from Moondyne Cave (**Figures 11v,vi**) and needle shaped crystals of sizes between 10 and 30 μm were recorded for enrichments from Mammoth Cave speleothems (**Figures 11vii,viii**). Some EPS formation was also noticed in a few sections. In all cases, the carbonate crystals were seen associated with bacterial cells indicating that they acted as nucleation sites, leading to their entrapment and formation of calcium carbonate. XRD analysis of the crystals identified various polymorphs of calcium carbonates in different sets (**Figure 12**). Calcite was seen as the predominant form in the Lake and Moondyne Cave enrichments (with Firmicutes) while vaterite was the major phase in the case of Proteobacteria dominated Mammoth Cave enrichments. No precipitates were seen in the control sets indicating that biotic process plays a crucial role in carbonate formation. The variations in carbonate polymorphs seen in the different communities could be a result of differing bacterial metabolic activities affecting dissolved inorganic carbon (DIC), and saturation index which could influence the type and properties of the calcium carbonate polymorphs (Rodriguez-Navarro et al., 2012; Dhami et al., 2013a; Okyay and Rodrigues, 2015). As microbial metabolic activity plays a role in determining the fate of the carbonate polymorph, this may explain the predominance of calcite formation with Firmicutes and vaterite formation with Proteobacteria as seen in the current study (Jimenez-Lopez et al., 2008; Jroundi et al., 2014; Zhu and Dittrich, 2016). A number of previous studies have reported the formation of biogenic carbonate crystals via bacterial strains isolated from cave environments (Cacchio et al., 2003; Rusznyak et al., 2012; Banerjee and Joshi, 2014). This work further supplements the hypothesis that biomineral production is a general phenomenon by different bacterial isolates and similar microbial activities might play an important role in the formation of speleothems in natural environments but still there is a lack of evidence of cause and effect. The associations between bacterial species, metabolic activities and their effects on mineral polymorphs need further studies. Advanced OMICS and radiolabelling techniques to identify active metabolic processes in speleothem formations from different climatic environments can be carried out in future studies. Our previous studies recorded the nanomechanical properties of different polymorphs of calcium carbonate crystals via ureolytic and carbonic anhydrase route wherein they varied in modulus from 36 ± 10 – 64 ± 2.7 GPa and hardness of 2.32 ± 0.15 – 3.92 ± 0.43 GPa; which are quite low compared to calcite rich cave speleothems of current study (Dhami et al., 2016). Further studies on nanomechanical properties of carbonate minerals synthesized by speleothem surface associated bacterial strains needs to be carried out in the future to investigate how these properties change at different time scales under cave simulated environments to provide deeper understanding of the bio-geo-chemical processes in nature. Though the generation of calcium carbonate biominerals has been successfully achieved in laboratory conditions but in comparison to their natural counterparts, their mechanical performance is much lower. In order to widen the scope of MICP technology for engineering applications, comprehensive analysis and optimization of physical and chemical conditions to copy mechanical properties of natural biomineralized materials in shorter time spams is required. This challenge demands researchers from diverse areas as microbiology, molecular biology, geology, material/civil engineers and chemists to work together and bridge the gap for production of sustainable bio-cement with properties of naturally biomineralized formations.

## Conclusion

In the current study, combined mineralogical, elemental, microbial, and nanomechanical characterization of different cave speleothems such as moonmilk, stalagmite, and stalactite has been conducted for the first time. Mineralogical and elemental analysis of cave speleothems varied from calcite, aragonite, dolomite, ankerite to silicates while micrographic analysis recorded differences in size, type, and shape of structures under different conditions. This analysis indicated the influence of geochemistry on mineralogical properties of different formations. Nanomechanical properties of these mineralized formations indicated that mineralogical signatures had profound effect on the mechanical properties. The moonmilk speleothem sample which was composed purely of calcite had higher strength properties compared to other speleothems indicating a connection between the two. On the other side, microbial diversity study of bacterial communities associated with the different cave speleothems specified that variations in assemblages were related to the mineralogy and geochemistry. Under *in vitro* conditions, microbial cultures associated with speleothem surfaces were enriched in nutrient rich conditions and all the biotic sets successfully precipitated calcium carbonate minerals. The generation of carbonate biominerals by cave speleothem associated bacterial communities in laboratory conditions support the hypothesis that similar processes may occur in nature. Even in lab conditions, noticeable variations in carbonate polymorphs were recorded highlighting the effect of microbial metabolism and species on carbonate mineralogy. Microbial diversity data under enriched conditions revealed significant variations in the assemblages from real field communities which highlighted the importance of physical and environmental conditions in selection of microbial communities under different conditions. Though the community structures and their metabolisms are selected by nutrient conditions and physico-chemical environments, they in return further affect the mineralogical and nanomechanical fate of the biominerals. Comparison of nanomechanical properties of naturally biomineralized formations with laboratory synthesized carbonate biominerals formed by MICP technology showed significantly higher performance of natural materials. In order to truly mimic the formation of such materials in laboratory conditions for engineering applications and synthesis of sustainable cements, further investigations combining geochemistry, mineralogy and next generation OMICS tools need to be carried out. Whether the performance of laboratory based biominerals formed harnessing different bacterial metabolic routes can be improved through optimization of physical and chemical conditions in shorter time spams; will help in determining the fate of MICP technology.

## Author Contributions

ND contributed by providing with concepts and ideas, fieldwork, experiments, and writing. AM contributed with concepts and ideas, fieldwork organization, data interpretation, and writing. EW contributed with microbiological data interpretation and writing.

## Conflict of Interest Statement

The authors declare that the research was conducted in the absence of any commercial or financial relationships that could be construed as a potential conflict of interest.

## Footnotes

1 https://github.com/lh3/seqtk
