# Supplementary material for: Microbial Diversity and Mineralogical-Mechanical Properties of Calcitic Cave Speleothems in Natural and in Vitro Biomineralization Conditions
Source: Front Microbiol. 2018 Feb 2;9:40. doi: 10.3389/fmicb.2018.00040 (PMC5810276; doi:10.3389/fmicb.2018.00040)
Supplement: Supplementary file 1 [file Table_1.DOCX]

*i. From original speleothems of Lake Cave, Moondyne Cave and Mammoth Cave*

**Table 1. Percentage OTUs from Bacterial 16S rRNA gene sequencing analysis at different levels**

**a. Phylum Level**

**b. Family Level**

**c. Genus Level**

*ii. From laboratory enriched conditions*

**a. Phylum Level**

**b. Family Level**

**c. Genus Level**

**Table 2. Heat map of dominant microbial communities associated with different speleothem enrichments at genus level**

| **Taxon** | Lake Cave moonmilk | Lake Cave stalagmite | Lake Cave stalactite | Moondyne Cave stalagmite | Mammoth Cave stalagmite | Mammoth Cave stalactite |
| --- | --- | --- | --- | --- | --- | --- |
| Unassigned | 0.00 | 0.02 | 0.01 | 0.00 | 0.00 | 0.02 |
| Bacillus (Firmicutes) | 87.40 | 12.33 | 99.57 | 67.99 | 6.60 | 0.16 |
| Brevibacillus (Firmicutes) | 0.00 | 0.01 | 0.01 | 20.10 | 0.00 | 0.00 |
| Paenibacillus (Firmicutes) | 0.96 | 5.54 | 0.00 | 0.09 | 8.44 | 0.75 |
| Planococcus (Firmicutes) | 0.17 | 80.77 | 0.00 | 0.00 | 2.02 | 0.00 |
| Lysinibacillus (Firmciutes) | 9.61 | 0.04 | 0.00 | 0.04 | 0.02 | 0.00 |
| Caulobacter (Alphaproteobacter) | 0.00 | 0.00 | 0.00 | 0.00 | 27.11 | 0.00 |
| Burkholderiales (Betaproteobacteria) | 0.00 | 0.00 | 0.00 | 0.00 | 47.99 | 7.56 |
| Pseudomonas (Gammaproteobacteria) | 0.00 | 0.01 | 0.03 | 6.75 | 0.00 | 72.86 |

Abundance scale (Green = High; Yellow = Medium; Red= Low)
